# Enhancing the Performance and Reliability of an Automotive Reed Sensor Through Spring Integration and Advanced Manufacturing

**DOI:** 10.3390/s25216778

**Published:** 2025-11-05

**Authors:** Umar Farooq, Valentina Bertana, Sergio Ferrero, Domenico Cantarelli, Luca Costa, Simone Bigaran, Luigi Costa, Luciano Scaltrito

**Affiliations:** 1 Chilab-ITEM Laboratory, Department of Applied Science and Technology (DISAT), Politecnico di Torino, Corso Duca degli Abruzzi 24, 10129 Turin, Italy; valentina.bertana@polito.it (V.B.); sergio.ferrero@polito.it (S.F.); 2 Department of Science and Technology Innovation (DISIT), Università del Piemonte Orientale, Viale Teresa Michel 11, 15121 Alessandria, Italy; 3 R&D Innovation Department, MISTA S.p.A, Via Roma 79/A, 14040 Asti, Italy; domenico.cantarelli@mista.it (D.C.); luca.costa@mista.it (L.C.); simone.bigaran@mista.it (S.B.); luigi.costa@mista.it (L.C.)

**Keywords:** additive manufacturing, automotive reliability, magnet alignment, reed sensors, sensor reliability, switching performance

## Abstract

Reed sensors play an important role in improving the safety, reliability, and efficiency of modern electric vehicles. Our study evaluates their performance by measuring the switching distance under five different configurations of a cylindrical magnet using a 3D-printed test fixture. Statistical analysis revealed that the right-shift-upward configuration yielded the best performance, significantly reducing the release distance. Building on this, a prototype housing was developed using Selective Laser Sintering with polybutylene terephthalate, and a stainless-steel spring was incorporated to enhance sensitivity and reliability. The spring integration reduced the activation distance to 2.3 mm, which is an improvement of up to 60%, and it also significantly improved the consistency of the results. These outcomes demonstrate a practical method for manufacturing more reliable reed sensors for automotive sensing technology.

## 1. Introduction

Modern sensor technologies play an important role in the advancement of electric vehicles (EVs) for improving efficiency, safety, and reliability [1,2,3]. Advanced EVs utilize a wide range of sensors including temperature sensors [4], pressure sensors [5], motion sensors [6], proximity sensors [7], and light sensors [8] for performance enhancement of different systems. Temperature sensors are important for thermal management, while pressure sensors are used in fuel and braking systems. Motion and proximity sensors are utilized in advanced driver-assistance systems (ADAS), which help in preventing collisions as well as in automated parking. Recent developments in sensor technologies and their integration with the Internet of Things (IoT) in the automotive industry have further improved innovative data processing capabilities [9,10].

Reed sensors have become increasingly important in automotive systems for their reliable operation and durability under extreme conditions [11]. A standard reed sensor comprises a hermetically sealed glass tube containing two ferromagnetic contacts separated by a small distance. These separated contacts are connected to complete the electric circuit when an external magnetic field of the required specific strength is introduced. This simple working principle allows rapid response, high insulation resistance, and stable performance in electric and hybrid vehicles to ensure safety and reliability in various applications. Moreover, various fabrication techniques have made it possible to manufacture more compact and robust reed switches for applications like liquid level sensing, and current monitoring in EVs [11,12,13].

Nevertheless, reed sensors encounter some challenges in practical applications. They are mainly vulnerable to high temperatures, vibrations, and electromagnetic interference, which can compromise both functionality and lifetime [13]. Likewise, mechanical wear and contact degradation may also lead to increased electrical resistance and raise the risks of failure of the sensor to perform its required function. In addition, vibrational forces can induce contact chatter and electrical noise, further reducing the reliability of the sensor [14]. Although reed switches remain cost-effective, their integration into advanced electronic systems is often limited. Moreover, a persistent gap between research findings and practical implementation still exists as numerous studies solely focus on theoretical modeling, as highlighted elsewhere [15]. These limitations emphasize the need for practical approaches to evaluate and enhance the performance, sensitivity, and reliability of reed sensors through improved design approaches, proper material selection, and utilization of advanced fabrication techniques.

To overcome these limitations, various approaches have been developed to improve the reliability and efficiency of reed sensors and switches. For instance, in some studies, the emphasis was on structural optimization, the use of high-performance materials, refined fabrication methods, and the implementation of noise reduction and error compensation techniques designed for various automotive operations [14,16,17]. Advances in materials, including transition metal dichalcogenides such as WS_2_ and MoS_2_, have yielded sensors with greater sensitivity, durability, and biocompatibility [17]. Researchers are also investigating graphene-based composites and conductive inks to expand active surface areas and enhance sensing capabilities [18]. Moreover, advanced polymers and composite housings can improve resistance to thermal and mechanical stress, making these sensors feasible for harsh automotive environments. Micro-electromechanical system (MEMS) technology has enabled the development of miniaturized, highly sensitive reed switches suitable for applications where space is limited [19]. Additive manufacturing, such as Fused Deposition Modeling (FDM) and Selective Laser Sintering (SLS), has further assisted in the fabrication of various prototypes with complex geometries and superior mechanical properties [20,21,22,23].

Our current study addresses the need for more reliable reed sensors through a two-stage approach. The primary objective was to systematically evaluate how magnet orientation influences switching behavior, moving beyond qualitative assessment to statistical validation. The secondary objective was to design, fabricate, and test a spring-integrated prototype that could mitigate the inherent sensitivity to alignment and vibration. By combining a detailed experimental study with advanced SLS manufacturing, this research provides a clear pathway for enhancing the robustness of reed sensors in demanding automotive environments.

## 2. Materials and Methods

Evaluation of the switching performance of the reed sensor was conducted in two phases. In the first phase, the effect of magnet orientation on switching distance was investigated. And, in the second phase, a prototype was developed to evaluate the improvement in switching performance, where incorporation of SS spring was carried out.

### 2.1. Materials

The reed switch used throughout both experimental series was a KSK-1A66-1530 model (Item no.: 2116601530), supplied by Standex MEDER Electronics. Each switch comprises two flexible ferromagnetic contacts (reeds) hermetically sealed within a glass tube. For the magnet orientation experiments, a cylindrical, iron-based magnet measuring 4mm in diameter and 10mm in height was used (Model: MFD100XXX040PAZ, CIBAS Group). The magnet was axially magnetized with a specified surface magnetic flux density of approximately 45 mT.

### 2.2. Methods

#### 2.2.1. Optimization Through Magnet Orientation

A custom test fixture was fabricated using black PLA TECH material via Fused Deposition Modeling (FDM) (3D-Dream Pro, 3D-Dream, Solbiate Arno, Italy). Key printing parameters included an extrusion temperature of 230 °C, a heated build plate temperature of 60 °C, and a fill density of 50%. The printer was equipped with a 0.4mm diameter brass nozzle and operated at a resolution of 0.1mm, with a layer thickness ranging from 0.1 to 0.3mm and a maximum modeling speed of 100mm/s. The system offered a build volume of 300 × 300 × 300mm and utilized 1.75mm diameter filament. These parameters were selected to produce a lightweight, rigid casing capable of facilitating rapid prototyping and ensuring precise, repeatable alignment of the magnet and sensor.

**Figure 1:**
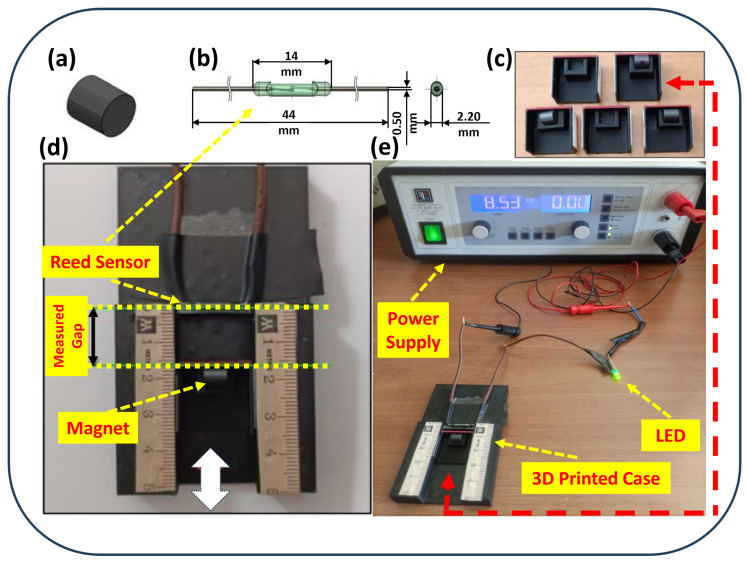
Experimental setup for initial reed sensor characterization: (**a**) cylindrical magnet, (**b**) reed sensor, (**c**) 3D-printed sliders for different magnet orientations, (**d**) FDM-printed PLA test fixture, (**e**) complete test assembly with measurement scale, power supply, and LED indicator.

**Figure 2:**
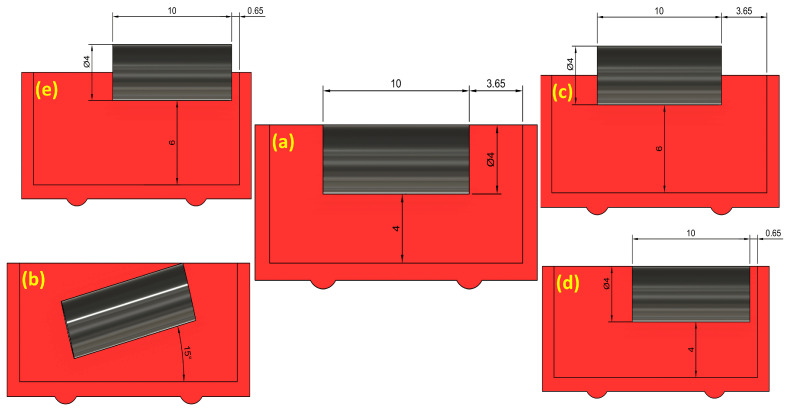
Schematic diagrams of the five tested magnet orientations relative to the reed sensor: (**a**) center-aligned, (**b**) center-angled, (**c**) center upward, (**d**) right-shift-aligned, (**e**) right-shift-upward.

Figure 1 illustrates the experimental setup, which comprises a reed switch, a magnet, and an integrated sliding mechanism (3D-printed case with a slider). A measurement scale was pasted on the case to precisely measure the switching distance between the magnet and the reed sensor. The reed switch was mounted horizontally in front of a slider containing a magnet. Five different sliders were fabricated, having cavities designed to orient the magnet in five different positions, as shown in Figure 2. These five different orientations represent the position of the magnet in the slider (Figure 1c) with respect to the position of the reed sensor, which can be seen in Figure 1d. The dimensions represented in the five different orientations are shown in “mm”.

Ten reed switches with straight terminals were tested under five magnet orientations: center-aligned, center-angled (15°), center-Upward (+2mm offset), right-shift-aligned (+3mm lateral offset), and right-shift-upward (+3mm, +2mm). Each reed sensor was connected to a power supply and an LED to visually observe its open or closed status during movement of the magnet placed opposite to it. For every orientation, both the “on” distance—at which the circuit is closed to light the LED—and the “off” distance—at which the circuit is open when the magnet is moved away from the sensor—are measured. Ten measurements were collected for each sensor and magnet orientation to ensure data reliability. While the manual operation of the slider could introduce minor variability, the use of a fixed measurement scale and ten repeated trials for each configuration ensured the reliability of the observed trends.

#### 2.2.2. Prototype Development with Integrated SS Spring

The prototype housing was fabricated from polybutylene terephthalate (PBT) using SLS (EOS Formiga P 110 Velocis, EOS GmbH, Krallling, Germany). This additive manufacturing process uses a laser to fuse powdered polymer particles layer by layer to build a solid structure with optimum precision. The purpose of selecting the SLS process and PBT material was to produce a single, compact, robust, and heat-resistant housing with intricate internal geometries to integrate all the components, including the magnet, reed sensor, and spring.

**Figure 3:**
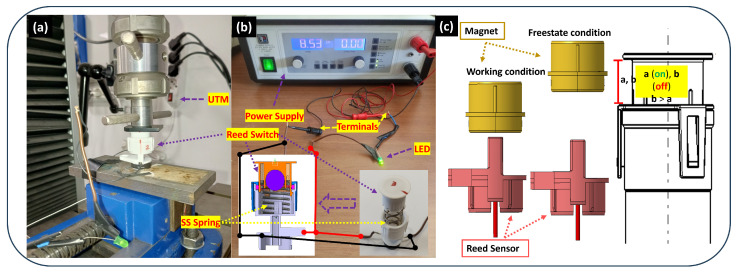
Switching performance of reed sensors with straight terminal spring-integrated prototype testing: (**a**) precise switching distance measurement using a Universal Testing Machine (UTM), (**b**) electrical connectivity verification setup showing the power supply and measurement instruments, and (**c**) schematic diagram of activation and release mechanism of reed sensor.

Figure 3 illustrates the test configuration for the spring-integrated prototype. The setup employed a Universal Testing Machine (UTM) (Galdabini S.p.a., Cardano al Campo (VA), Italy) to ensure precise control, along with a dedicated power supply. The reed switch and spring assembly were fixed within the SLS-produced PBT housing, which offered greater mechanical strength and thermal stability than the earlier FDM-printed case. This choice better replicated the conditions encountered in actual automotive environments. The precision of the SLS process ensured that internal cavities held the components securely, minimizing any movement that could affect results. The power supply energized the circuit, and an LED indicator was connected to signal switch activation (circuit closure). Voltage and current readings (e.g., 8.53 V, 0 A) were monitored on the power supply display.

The core objective of this experiment was to quantify the spring’s effect on the switch’s “on” and “off” parameters. This entailed a systematic evaluation under varied conditions, focusing on two spring tip orientations (0° and 90°) relative to the reed sensor. A total of twenty reed sensors were tested: ten with straight terminals and ten with bent terminals. The “on” and “off” distances, indicative of the magnetic proximity needed for circuit closure and opening, were meticulously recorded. The experimental design examined the impact of both spring orientation and sensor integration method (straight vs. bent terminals) on switching performance. The magnet orientation was maintained in a center-aligned position for all tests in this series. Each configuration was tested multiple times, and average values alongside standard deviations were calculated to rigorously assess consistency and operational reliability.

#### 2.2.3. Data Collection and Analysis

For both experimental series, the “on” and “off” distances were recorded. The “on” distance is defined as the point where the magnetic field strength is sufficient to attract the reeds and close the electrical contact, illuminating the LED. Conversely, the “off” distance is the point at which the reeds separate and the circuit opens upon removal of the magnetic field. Data was gathered from multiple reed switches across all tested conditions. The resulting dataset was analyzed to compute average values and standard deviations, providing critical insights into the sensitivity, reliability, and durability of the switches under each configuration. The mechanism of the reed switch in the working (on) and free-state (off) conditions is illustrated in Figure 3.

## 3. Results and Discussions

Our experimental findings reveal that both magnetic field orientation and the integration of a spring mechanism substantially influence the switching distances of reed switches, confirming our initial hypotheses regarding performance optimization.

### 3.1. Effect of Magnet Orientation on Switching Distances and Physical Interpretations

**Figure 4:**
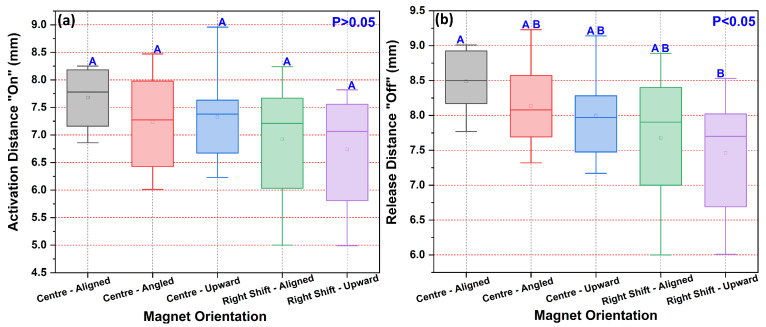
Switching distances for five magnet orientations. Letters indicate statistical groupings for (**a**) “On” (one-way ANOVA, *p* > 0.05) and (**b**) “Off” distances (one-way ANOVA, *p* < 0.05). Data are mean ± SD (n = 10).

The initial investigation focused on how magnet orientation affects the switching behavior of a standard reed sensor. The results, summarized in Figure 4, show a clear trend of five different magnet configurations where the magnet was shifted rightward and upward consistently yielded the shortest switching distances. Angled orientations produced on distances of 6.0–8.5 mm and off distances of 7.3–9.2 mm. Vertical displacement of the magnet while maintaining center alignment resulted in on distances of 6.2–8.9 mm and off distances of 7.2–9.6 mm. Right-shifted configurations exhibited even greater variability, with aligned right shifts yielding on distances of 5.0–8.2 mm and off distances of 6.0–8.9 mm, while right-upward shifts produced on distances of 5.0–7.9 mm and off distances of 6.0–8.5 mm. The right-shift-upward orientation performed best, with mean activation (“on”) and release (“off”) distances of approximately 7.1 mm and 7.6 mm, respectively. In contrast, the center-aligned configuration required the magnet to be closest, with the longest average release distance.

To determine the statistical significance of these trends, a one-way ANOVA with Tukey’s post hoc test was performed. The analysis revealed that the variation in activation (“on”) distances across the five orientations was not statistically significant (F(4,45) = 1.86, *p* = 0.135). However, a statistically significant effect was found for the release (“off”) distances (F(4,45) = 3.29, *p* = 0.019). The post hoc analysis assigned the “off” distances to statistical groups “A” and “B”, confirming that the right-shift-upward (Group B) configuration had a significantly shorter release distance than the center-aligned (Group A) orientation.

This finding indicates that magnet orientation has a definitive impact on the sensor’s release behavior, which is critical for ensuring a clean circuit break. The fundamental operating principle is that the ferromagnetic reeds inside the glass capsule close the electrical circuit when the external magnetic force exceeds their inherent spring stiffness. This magnetic force is a direct function of the strength and spatial gradient of the magnetic field at the location of the reed contacts. Our results can be interpreted by considering how each magnet orientation alters this field. In the center-aligned configuration, the magnetic field is symmetric but must act over the full gap between the magnet face and the sensor, resulting in the longest switching distances. In case of the right-shift-upward configuration (On: 7.1 mm, Off: 7.6 mm), shifting the magnet laterally and vertically likely brings one of its poles (North or South) significantly closer to the reed blades. This creates a localized region of higher field strength and a steeper field gradient, intensifying the magnetic force on the reeds and allowing them to close at a greater distance from the magnet’s center of mass. On the other hand, the center-angled and center-upward orientations create an asymmetric field that may not couple as efficiently with both reeds simultaneously, leading to intermediate and more variable switching distances. This analysis confirms that precise magnet alignment is not just a practical concern but is rooted in the fundamental physics of magnetic interaction, which is crucial for optimizing sensor packaging in constrained automotive environments.

The variations in the switching distances of the sensor highlight the limitations of operational consistency depending on the position and orientations of the magnet. In addition, by replacing the magnet with a magnet of a different magnetic flux density, it will further affect the sensitivity and operational efficiency of the reed sensor. Similarly, the material of the fixture/case used to evaluate the performance of the reed sensor plays a key role. The FDM-printed PLA housing played an important role in maintaining measurement accuracy. Its rigid, lightweight construction and simple design improved the optimization process. The internal structure provided adequate rigidity to secure the alignment of components, including the sensor and magnet. This manufacturing approach not only reduced measurement errors but also supported cost-effectiveness and rapid prototyping, and allowed for testing using all orientations of the magnet.

### 3.2. Effect of Integrated Spring Mechanism on Switching Distances

**Figure 5:**
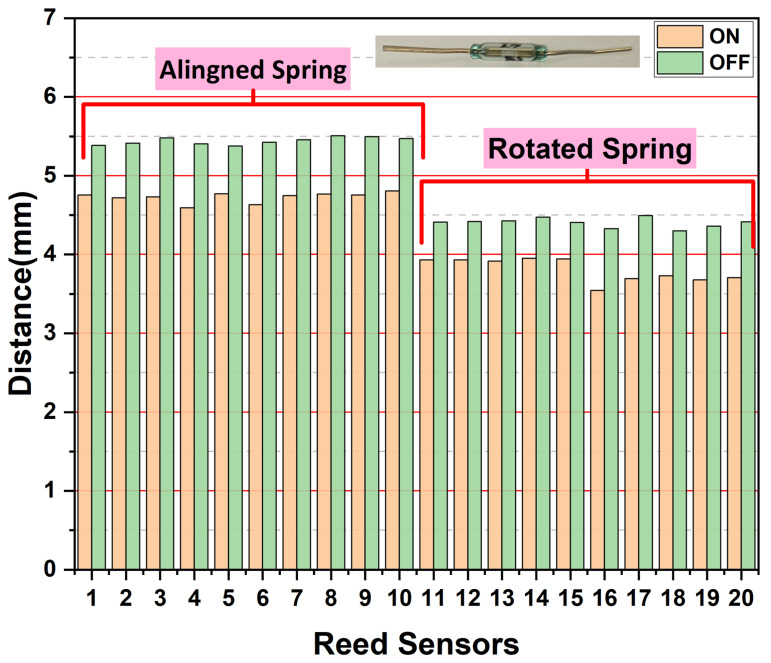
Switching performance of reed sensors with straight terminals, comparing aligned (0°) and rotated (90°) spring orientations.

**Figure 6:**
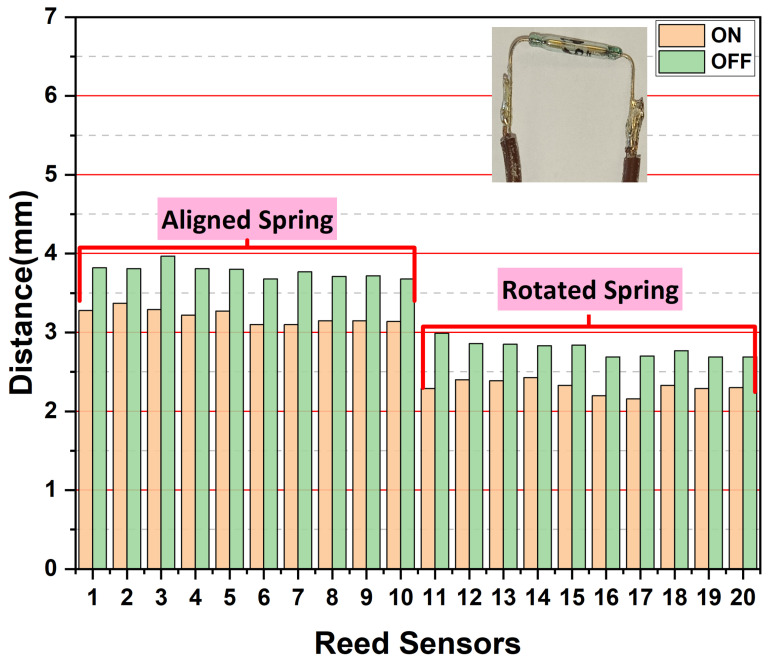
Switching performance of reed sensors with bended terminals, comparing aligned (0°) and rotated (90°) spring orientations.

To improve performance and reduce sensitivity to magnet positioning, a spring-integrated prototype was developed. The assembly, housed in a cylindrical SLS-fabricated PBT casing, was tested using a UTM for precise measurement (Figure 3). The prototype testing allowed systematic investigation of how spring orientation (0°, 90°) and terminal geometry (straight and bent) influence the switching behavior of the reed sensor , as indicated in Figure 5 and Figure 6.

Incorporation of the spring mechanism prominently improved the switching performance of the reed switch, as shown in Figure 5 and Figure 6. In the case of the straight terminals of the reed sensor, with spring edge alignment, the switch activated at an average distance of 4.7 mm and deactivated at 5.4 mm, demonstrating exceptional consistency (σ = 0.05 mm). Rotating the spring edge with straight terminals reduced these distances to 3.8 mm (on) and 4.4 mm (off), though with slightly increased variation (σ = 0.14 mm). The most pronounced improvement occurred with bent terminals and aligned spring, where distances decreased to 3.2 mm (on) and 3.8 mm (off) while maintaining excellent consistency (σ < 0.1 mm). As quantified in Table 1, the best-performing configuration (bent terminals with rotated spring) reduced the activation distance to just 2.3 mm, which represents an improvement of up to 60% compared to the best non-spring configuration. This enhancement can be understood through a mechanical analysis of the spring’s role. These findings clearly establish that both terminal geometry and spring orientation critically influence switching sensitivity and consistency.

Although reed switch performance is essentially affected by the position of the magnet with respect to the reed sensor, the results show that adding a spring mechanism between the magnet and the sensor promotes more uniform operation and reduces switching distances, thus making the device more responsive and predictable. This improvement can be further understood through a mechanical analysis of the spring’s role. Firstly, the spring introduces a mechanical preload on the assembly. By pressing against the sensor housing, it creates a counter-force that the magnetic attraction must overcome. This preload means less physical displacement is required to achieve the magnetic force needed for contact closure, directly explaining the substantial reduction in switching distance. Secondly, the spring is hypothesized to act as a mechanical vibration damper. The significantly improved consistency across all tests, evidenced by the low standard deviations (e.g., σ ≤0.1 mm for bent terminals) in Table 1, strongly suggests enhanced mechanical stability. The spring adds compliance to the system, which is expected to absorb energy from shocks and vibrations, thereby isolating the sensitive reed contacts and preventing false triggering (chatter). It is important to note that direct validation of this anti-vibration performance under dynamic loads is a key objective for future work. In addition to these sensitivity gains, the housing fabricated from PBT using SLS also provides superior thermal and mechanical resistance over conventional materials. The SLS process produced a precise, seamless enclosure that securely held all components in their respective positions to avoid internal movements that could lead to performance inconsistencies.

**Figure 7:**
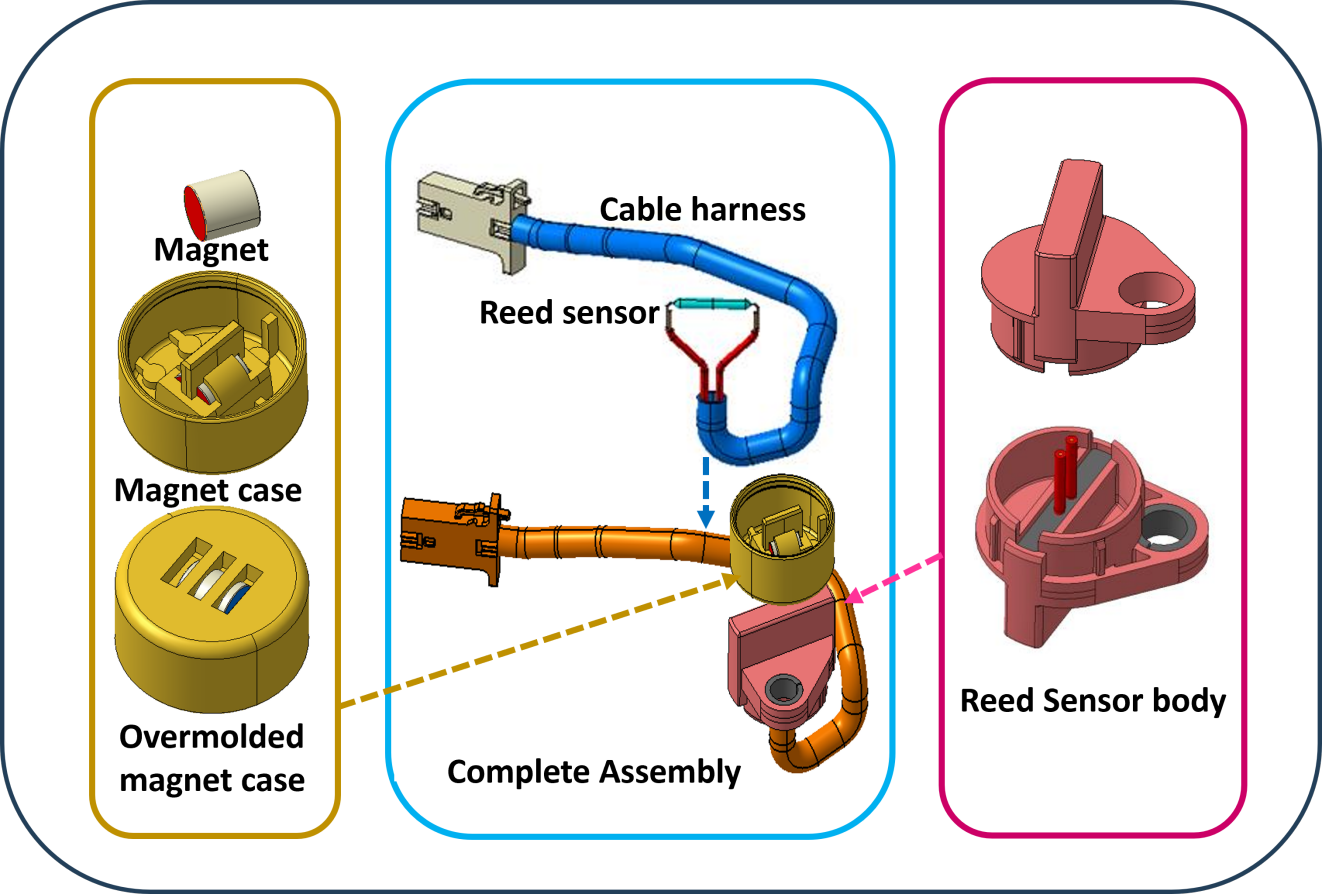
Exploded view and final assembly of the automotive reed sensor prototype, showing the magnet, magnet case, overmolded magnet case, PBT housing, reed sensor, and cable harness.

Figure 7 shows the fully assembled sensor unit. Its compact design incorporates secure wiring and protective housing to ensure consistent operation in required automotive operations. It could be installed for position sensing in trunks, doors, hoods, etc., as well as for monitoring gear selectors and pedals. The suitability of this sensor lies in its non-contact principle of operation, which minimizes mechanical wear and, ultimately, enhances its operational lifespan. Additionally, the housing material (PBT) effectively shields the internal components from abrupt environmental challenges such as vibration, moisture, dust, and extreme temperatures to ensure consistent performance.

## 4. Limitations and Future Work

While our study shows significant performance improvements, certain limitations should be acknowledged to contextualize the findings. The testing was conducted under quasi-static conditions. The proposed vibration-damping effect of the spring, while based on sound mechanical principles and supported by improved consistency, requires future validation through standardized dynamic vibration and shock tests. The manual measurement method in the initial magnet orientation study is effective in identifying clear trends, but it introduces a source of variability to some extent. Future work will include dynamic testing, long-term environmental durability studies (thermal cycling, humidity) and a direct performance comparison with leading commercial reed sensor units to fully benchmark the proposed design.

## 5. Conclusions

This study shows that incorporating a spring mechanism and utilizing advanced manufacturing significantly improves reed sensor performance for automotive applications. We first systematically showed that magnet orientation drastically affects switching distance, with a right-shift-upward configuration yielding the best baseline performance. The subsequent integration of a stainless-steel spring within an SLS-fabricated PBT housing reduced the activation distance to as low as 2.3 mm, an improvement of up to 60%, and greatly enhanced operational consistency. The spring functions by providing a mechanical preload and is hypothesized to dampen vibrations. The use of advanced manufacturing techniques, including Fused Deposition Modeling (FDM) with PLA and Selective Laser Sintering (SLS) with PBT, for manufacturing of housing structures further assists in achieving sustainability goals, rapid optimization, and qualification of prototypes for final application. This novel approach of combining magnet optimization, spring integration, and advanced manufacturing provides a practical and effective pathway to more reliable reed sensors.

## Figures and Tables

**Table 1:** Switching performance of spring-integrated prototypes.

Configuration	Spring	ON Distance	OFF Distance
	Orientation	(mm)	(mm)
Straight terminals	Aligned (0°)	4.7±0.05	5.4±0.05
Straight terminals	Rotated (90°)	3.8±0.14	4.4±0.14
Bent terminals	Aligned (0°)	3.2±0.10	3.8±0.10
Bent terminals	Rotated (90°)	2.3±0.10	2.7±0.10

## Data Availability

Data is contained within the article.
